# PUTTING IT TOGETHER: THE COMPLEMENTARY EFFORTS OF PUBLIC HEALTH AND AGING TO ADDRESS DEMENTIA

**DOI:** 10.1093/geroni/igac059.277

**Published:** 2022-12-20

**Authors:** Nora Super

**Affiliations:** Milken Institute Center for the Future of Aging, Santa Monica, California, United States

## Abstract

The Public Health Centers of Excellence are part of a larger effort by the Centers for Disease Control and Prevention (CDC), through the BOLD Infrastructure for Alzheimer’s Act and the Healthy Brain Initiative, to advance cognitive health and dementia as public health issues – and to expand the public health response to the Alzheimer’s crisis. These efforts are designed to complement, not supplant, the long-standing work of the aging community to help people with dementia and their families. This presentation will discuss how the new and expanded public health work corresponds with the efforts of the aging community and will illustrate the unique roles of the public health and aging communities in the fight against Alzheimer’s. In addition, the presentation will review initiatives – such as the Milken Institute’s Alliance to Improve Dementia Care – that seek to bring both communities together on a common dementia agenda.

